# Intravital 3-photon microscopy of leukocyte dynamics in the splenic white pulp

**DOI:** 10.1016/j.crmeth.2026.101609

**Published:** 2026-09-25

**Authors:** Dominik Schienstock, Sapna Devi, Scott N. Mueller

**Affiliations:** 1 Department of Microbiology and Immunology, The University of Melbourne, The Peter Doherty Institute for Infection and Immunity, Melbourne, VIC, Australia

**Keywords:** 3-photon microscopy, intravital imaging, image analysis, spleen, white pulp, T lymphocytes, leukocyte migration

## Abstract

Intravital imaging has been instrumental in uncovering the complex behaviors of immune cells during immune responses against viruses and tumors. The spleen is a major hub for systemic immune responses and immune memory. However, imaging of the spleen remains technically challenging, limiting our understanding of the dynamics of cellular interactions within specialized spleen niches, including the white pulp. Here, we present a method to stabilize the spleen for intravital imaging and to capture cell dynamics of the white pulp using 3-photon microscopy. We detail how to process images, quantify cell migration, and integrate behavior with structural information, revealing stromal guidance of T cell migration within deep T cell zones. This method of intravital 3-photon imaging of splenic white pulp supports in-depth investigation of cell dynamics and interactions in health and disease.

## Highlights

• We develop a method enabling stable intravital 3-photon microscopy of the mouse spleen
• Reduced light scattering of 3-photon imaging enables white pulp visualization
• Cellular behaviors are revealed in deep T cell zones
• SHG and THG imaging reveals tissue structures that guide leukocyte migration

## Motivation

Imaging of leukocyte dynamics in white pulp, which are typically located several hundred microns from the spleen capsule, is hampered by the limited depth of imaging achievable by 1- and 2-photon imaging due to light scattering of the blood-rich red pulp. The anatomical location of the spleen also presents difficulties for intravital imaging due to movement induced by respiration. To overcome this, our method introduces a platform to stabilize the surgically exposed mouse spleen for deep 3-photon imaging of leukocytes in the white pulp. Our workflow enables 4D imaging of labeled cells, label-free imaging of spleen structure, and advanced image analysis methods to dissect cellular behaviors in splenic white pulp.

## Graphical abstract

**Figure:**
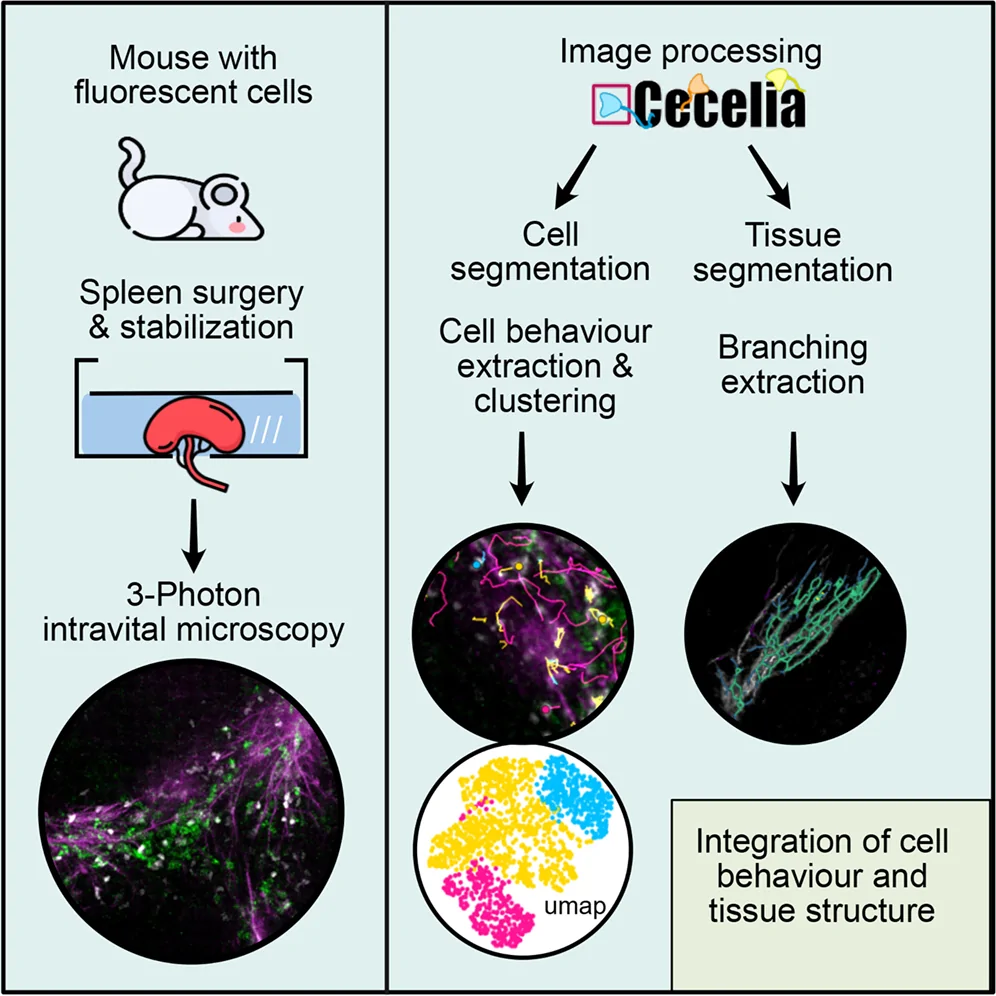

## Teaser

Schienstock et al. present a technical framework using surgical preparation of the mouse spleen, intravital 3-photon microscopy, and quantitative image analysis to interrogate *in vivo* leukocyte dynamics in the splenic white pulp. This method facilitates the investigation of cellular behaviors that underpin spleen biology in health and disease.

## Introduction

Intravital imaging is on the forefront to uncover cell migration patterns within tissues that underpin effective immune responses. The spleen is the main organ where primary responses against systemic infections are initiated and sustained.^1^^,^^2^ The functional niches of the spleen are the red pulp, where red blood cells are located, and the white pulp, where the B and T cell zones are located. Imaging of the spleen is challenging due to the thick capsule, erythrocyte-rich red pulp, and diverse structural and cellular components, which result in complex tissue scattering. Unlike imaging of the lymph node^3^^,^^4^^,^^5^ where T cells in the interfollicular space (up to 250 μm in depth) can be readily visualized and immune responses can be analyzed, imaging immune responses in the spleen has been restricted to shallow (∼100 μm) regions of interest using 1- and 2-photon microscopy.^6^^,^^7^^,^^8^ Although 2-photon imaging was successfully used in one study to assess lymphocyte movement in the spleen,^6^ it is challenging to locate a white pulp amenable for imaging, as these typically reside at least 200 μm from the capsule in mice.

To achieve deeper imaging of the mouse spleen, we used 3-photon microscopy and designed a specialized imaging platform. 3-Photon microscopy uses short-wavelength infrared light (1,250–1,750 nm), which has a longer wavelength than that used for 2-photon microscopy (typically 800–1,100 nm) and penetrates deeper into tissues due to reduced scattering. Intravital 3-photon imaging is well established to investigate neuronal activity in the mouse brain^9^^,^^10^^,^^11^ and has been utilized to extract lymphocyte dynamics deep in the popliteal lymph node.^12^ Given the depth advantage of 3-photon microscopy, we developed a method for imaging the splenic white pulp. A further benefit of 3-photon excitation is the capacity for label-free imaging of tissue structures without a requirement for exogenous labels, fluorochromes, or antibodies using second and third harmonic generation (SHG and THG, respectively).^11^^,^^12^^,^^13^

To achieve stable intravital imaging of the mouse spleen, we developed a custom-made mounting platform that isolates the spleen from the abdomen and reduces sample motion induced by respiration. We demonstrate the use of this imaging method to visualize leukocyte migration in the splenic white pulp and apply image analysis tools to interrogate cellular behaviors. We further demonstrate the capacity to image collagen-rich structures and blood vessels in the central T cell zone using SHG and THG imaging and use this to reveal how the tissue microarchitecture guides local lymphocyte directional migration.

## Results

### A platform for stable intravital imaging of the spleen

Stable intravital imaging of the splenic white pulp to capture leukocyte dynamics remains highly challenging. We aimed to develop a method to aid researchers in addressing experimental questions in the spleen, from surgical preparation and mounting of the spleen and intravital imaging through to image processing and cellular behavioral analysis (Figure 1A). We built a platform to hold animals during imaging that consists of a custom-made metal restrainer (Figures 1C andS1). Using stainless steel sheeting, screws, and springs, the platform was designed to be robust and replicable. A plate with a 2-cm diameter hole in the center enabled the isolation and stabilization of the exteriorized spleen (Figures 1B–1D). The platform was adjusted with spring-loaded screws to accommodate a range of animal sizes (Figure S1). The mice were anesthetized, and skin of the flank over the abdomen was shaved before a 0.5-cm incision was made through the skin and the peritoneum (Figure 1Di–iv; Video S1). Using a plastic tissue culture dish with a hole cut in the bottom placed on the platform over the incision site, the spleen was partially exteriorized into the dish and further stabilized with agarose placed around the tissue (Figure 1Dv–xii). The imaging platform and mouse could then be transferred directly onto a microscope stage (Figure 1E), where the animal remains anesthetized, is kept warm using a heat pad under the stage, and can be monitored regularly throughout the experiment.

**Figure 1:**
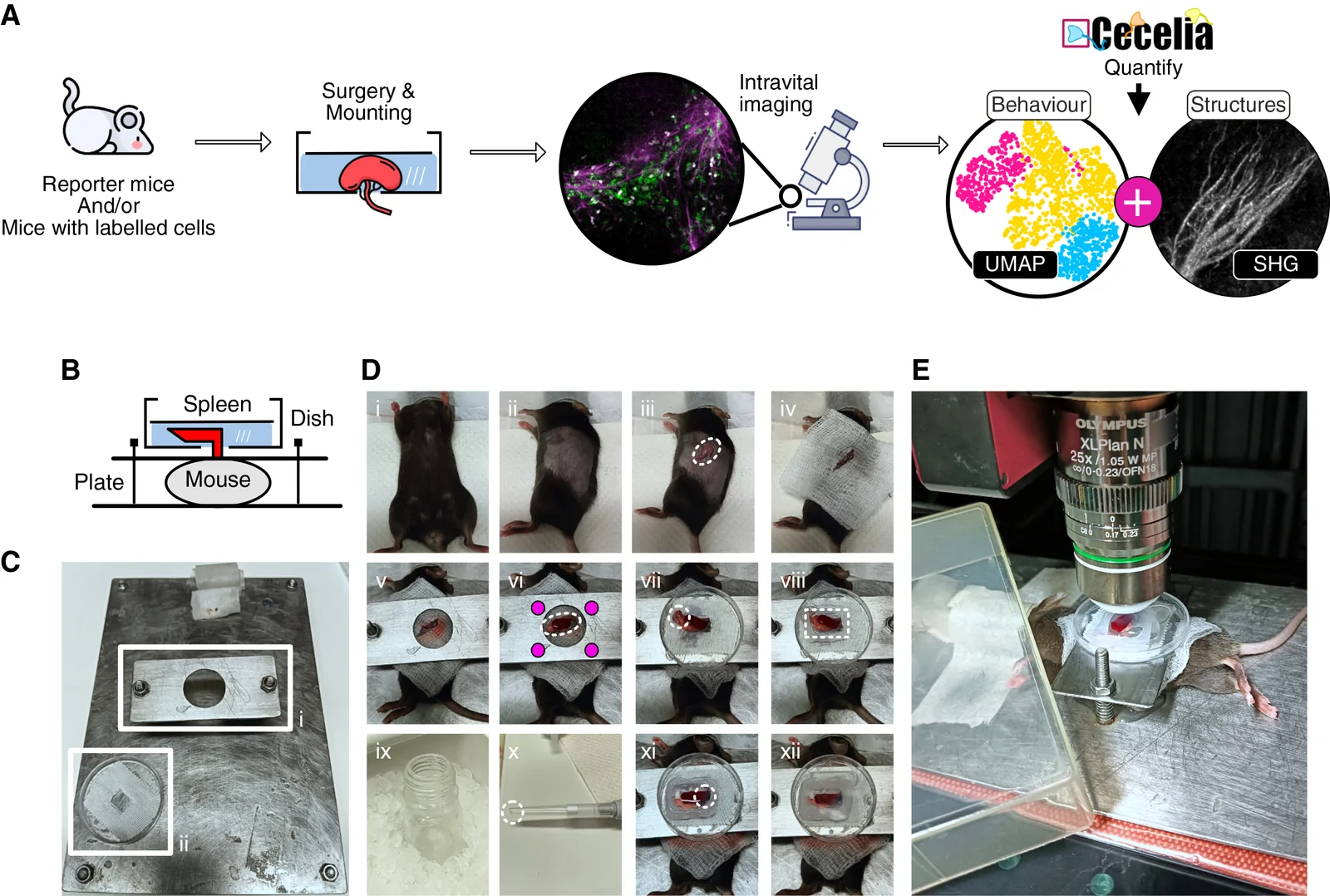
Mouse spleen preparation for intravital imaging (A) General workflow for intravital 3-photon imaging of mouse spleen. Transgenic reporter mice or mice with adoptively transferred fluorescently labeled T cells were surgically prepared to expose and stabilize the spleen before intravital 3-photon imaging. Images are subsequently processed and analyzed using a custom image analysis pipeline. (B) Diagram of the mounting setup to expose the spleen on an imaging dish. (C) The spleen imaging platform (i), and the prepared imaging dish (ii). Detailed description of dimensions can be found in Figure S1. (D) Step-by-step visualization to surgically prepare and stabilize the spleen for intravital imaging (i–xii). (E) Prepared spleen setup on the microscope stage. This figure has been designed using resources from Flaticon.com.

Video S1. Step-by-step visualization of spleen preparation for intravital imaging as depicted in Figure 2C, related to Figure 1

With our spleen mounting method, approximately one-third of the spleen or more can be visualized, as the procedure enables a sufficiently large region of the diaphragmatic surface of the spleen to be horizontally positioned relative to the microscope objective. Previous strategies have relied on compressing the mice significantly to create a contact surface between the coverslip and the spleen^6^ or applying glue.^8^ Our method exteriorizes the spleen and isolates the organ from the mice. This reduces the motion caused by the animal’s respiration and improves spleen stability, which is essential for 3-photon imaging because tissue drift increases the risk of focal plane heating^13^ and consequent tissue damage.

### 3-Photon microscopy enables reliable imaging of the splenic white pulp

To image the spleen, we used a customized Thorlabs Bergamo II multi-photon microscope system with super broadband optics for imaging from 680 to 1,900 nm. A Class 5 White Dwarf dual output 3-photon laser with tunable 1,300 and 1,700 nm laser lines was used, as well as a Coherent Chameleon Discovery laser for comparative 2-photon imaging. We imaged XCR1-venus mice, which express the yellow fluorescent protein under the *Xcr1* promoter in conventional type 1 dendritic cells (DCs). Prior to imaging, the mice were injected with 4 × 10^6^ tdTomato-expressing lymphocytes isolated from ubiquitous tdTomato mice.^14^ We compared intravital 2-photon and 3-photon imaging of the spleen in the same mice (Figure 2). 2-Photon imaging at 920 nm (80 MHz pulse repetition rate) permitted imaging of the spleen to a depth of approximately 100 μm from the capsule, enabling visualization of the red pulp (Figure 2A). In contrast, 3-photon imaging at 1,300 nm and 2 MHz enabled imaging to at least 250 μm, including white pulp regions. SHG imaging of collagen in the spleen was also improved using 3-photon excitation. In particular, the collagen-rich stromal network in T cell zones was strongly excited and enabled reliable identification of white pulp (Figures 2A and 2B).

**Figure 2:**
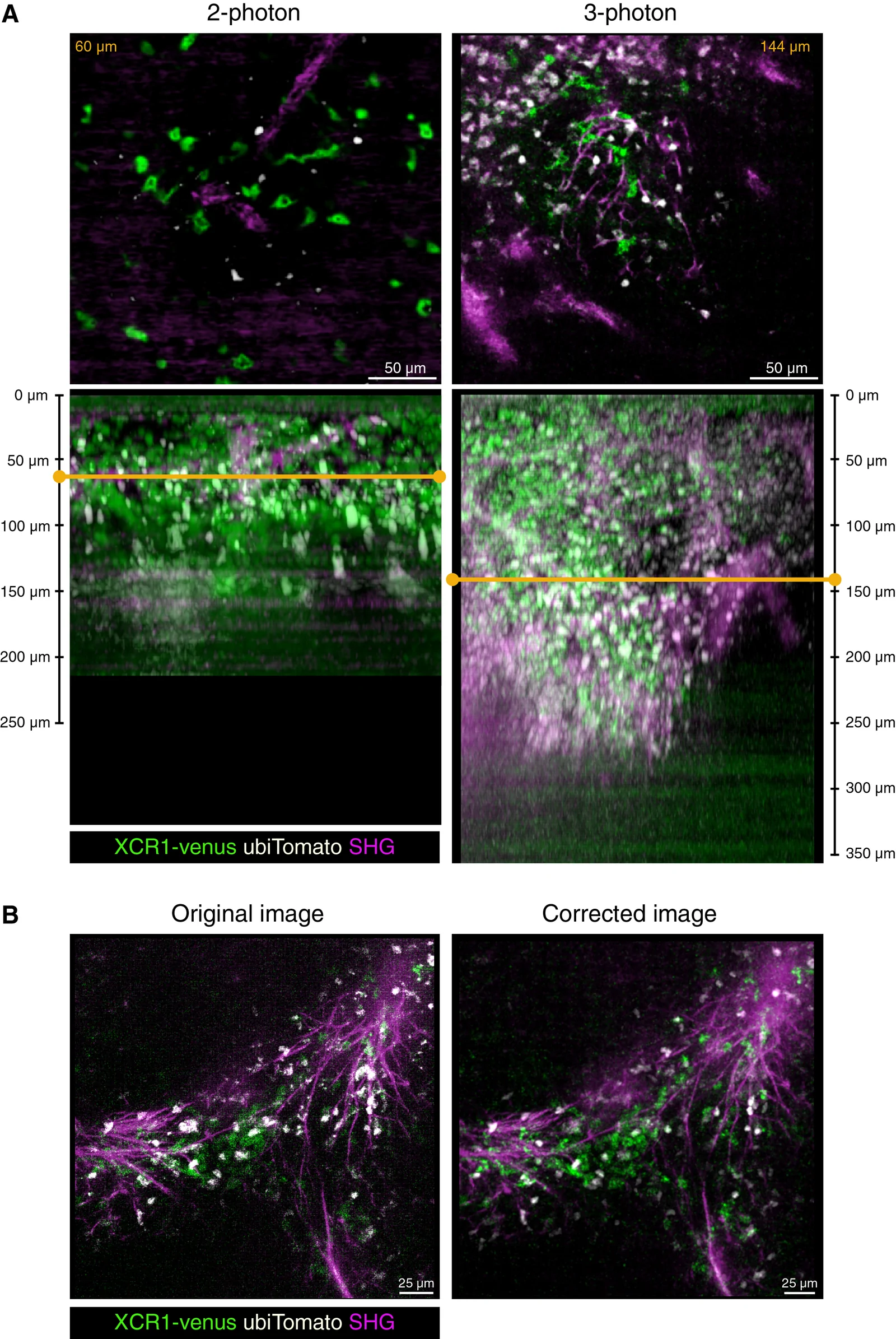
Intravital multi-photon microscopy of mouse spleen (A) Comparison of 2-photon and 3-photon microscopy of the same imaging region of XCR1-venus mice with adoptively transferred lymphocytes from ubiquitous tdTomato (ubiTomato) mice. Upper images show the *x*-*y* orientation, and below images show the *x*-z orientation, with the orange line indicating the z plane of the upper image. Images were processed using Cecelia, as described in STAR Methods. (B) Comparison between original and processed images, as described in STAR Methods.

The slow-pulse repetition rate of the 3-photon laser limits the acquisition speed, and thus the imaging volume captured during an acquisition time of 30 s was lower than that under 2-photon imaging (typically 13 frames 3 μm apart in 30 s with 2× frame averaging with 3-photon versus 50 frames 2 μm apart in 20 s with 4× frame average with 2-photon). Although imaging is often improved by frame averaging, this also extends the imaging time, further restricting the number of z planes that can be captured. Thus, to improve image quality, we performed post-processing by denoising with Cellpose^15^ within our image analysis framework Cecelia^16^ (Figure 2B; Video S2). This reduced noise and inhomogeneity within frames, reducing bright-intensity pixels and increasing low-intensity pixels. We then performed autofluorescence and drift correction on the images with Cecelia. This processing is required because multi-photon images often have autofluorescence and drift due to animal breathing.

Video S2. Original and corrected 3-photon movies Images were processed using the denoising algorithm in Cellpose and corrected for autofluorescence and drift, related to Figure 2

### 3-Photon imaging of lymphocyte dynamics in the splenic white pulp

To investigate lymphocyte migration and behavior within the spleen, we imaged adoptively transferred lymphocytes in the red pulp and white pulp. Lymphocytes enter the spleen via the marginal sinuses and exit the spleen via the red pulp but spend most time in the white pulp where interactions with DCs can facilitate priming to pathogen antigens.^2^ Accordingly, there were fewer lymphocytes in the red pulp, and as previously observed using 2-photon microscopy,^6^ these cells migrated slower than cells in the white pulp (Figures 3A and 3B; Video S3). We then compared lymphocyte dynamics in the spleen with that in lymph nodes, using intravital 3-photon imaging. As previously observed,^12^ cells migrating in the lymph nodes migrated at an average velocity of 10 μm/min, whereas lymphocytes migrating in the splenic white pulp migrated with an average velocity of 5 μm/min (Figure 3B).

**Figure 3:**
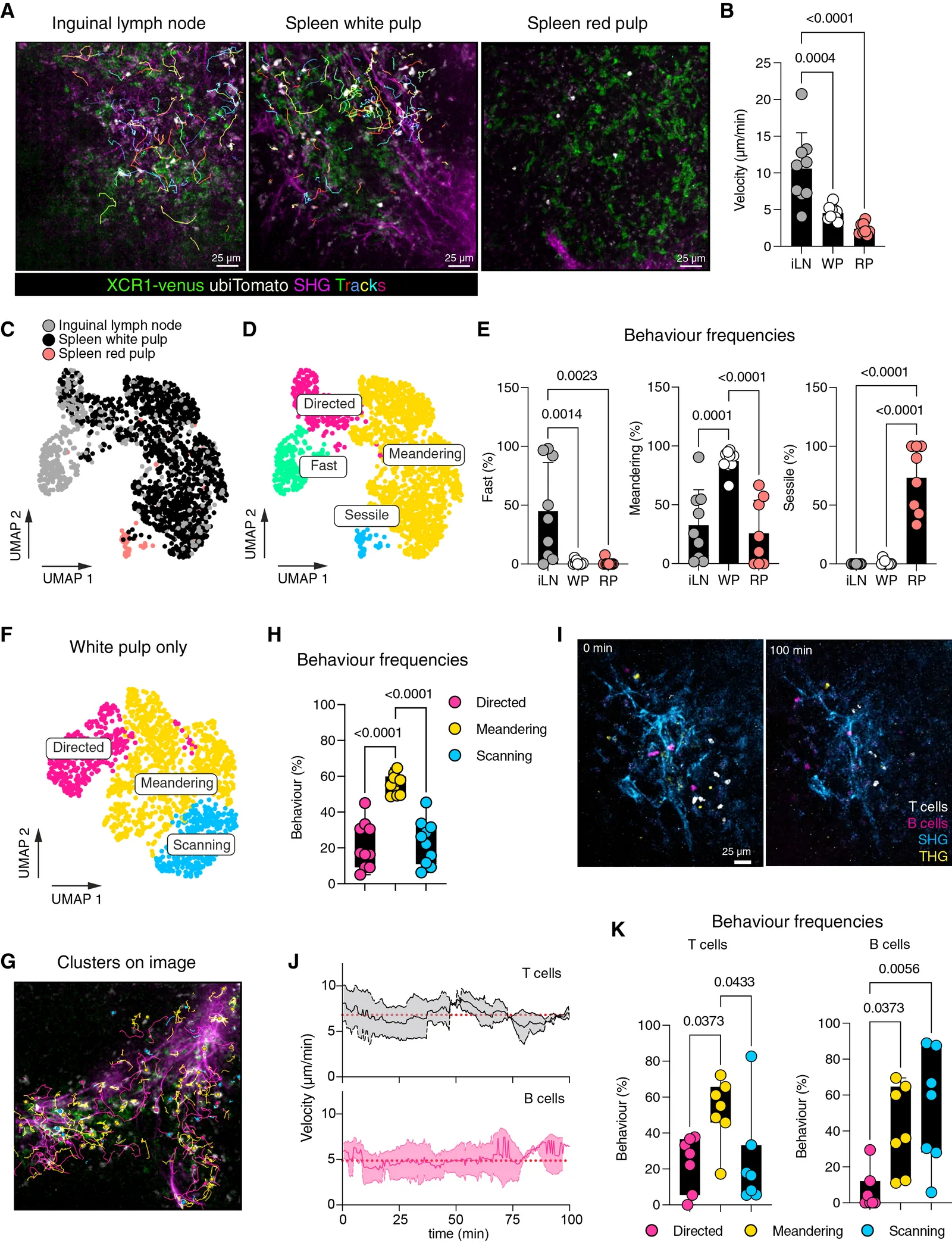
Intravital 3-photon imaging of lymphocyte cell dynamics in the splenic white pulp, red pulp, and lymph node (A) Processed and tracked images from inguinal lymph node (iLN) (*n* = 4 mice, 2–3 videos each), as well as splenic white pulp (WP) (*n* = 5 mice, 1–3 videos each) and red pulp (RP) (*n* = 4 mice, 1–3 videos each). (B) Velocity compared between tissue locations. One-way ANOVA with Tukey’s multiple-comparison test. (C and D) Resulting uniform manifold approximation and projection (UMAP) based on Leiden clustering using HMM, HMM transitions, and whole-track measurements (shown in Figure S2A). UMAP projections demonstrate the tissue source (C) and behavioral clusters (D). (E) Behavioral frequencies of the identified clusters. One-way ANOVA with Tukey’s multiple-comparison test. (F) UMAP from white pulp-only analysis. (G) Identified clusters on the representative image. (H) Comparison of behavioral frequencies across images. One-way ANOVA with Tukey’s multiple-comparison test. (I) 3-Photon imaging of the splenic WP in mice with transferred polyclonal GFP^+^ T cells (white) and tdTomato^+^ B cells (magenta). Representative images from the start of imaging (0 min) and after 100 min of continuous imaging, with images acquired every 30 s. Second harmonic generation (SHG, cyan); third harmonic generation (THG, yellow). (J) Velocity over the time period of imaging for T and B cells imaged in the WP. *n* = 4 mice, 7 videos. Dotted lines represent the average velocity of all cells imaged. (K) Behavioral frequencies of the identified clusters. One-way ANOVA with Tukey’s multiple-comparison test. All quantification data are represented by mean ± SEM.

Video S3. 3-Photon imaging of inguinal lymph node as well as splenic white and red pulp, related to Figure 3

To further interrogate the behavior of migrating cells in the white pulp, we performed an analysis of cellular behavior based on hidden Markov models (HMMs) and unsupervised clustering.^16^ Because unsupervised and semi-supervised trajectory-analysis tools such as Louvain and *k*-means do not take time-resolved information into account, we used HMM states to consider the time-dependent sequence of morphology and movement changes. We first determined the sequence of behavior within each track and then utilized the transitions between these behavioral sequences. The percentage of HMM states and their transitions were used to aid unsupervised Leiden clustering. We extracted HMM states after tracking the cells to describe the behavioral modes between lymph nodes and splenic white and red pulp (Figure S2A). Whole-track measurements and HMM states and their transitions were used for Leiden clustering (Figure S2A; Figures 3C–3E). We defined 4 cell behaviors between these three locations: fast (very high speed and low angle), directed (high speed and low angle), sessile (low speed), and meandering (intermediate speed and high angle) (Figure 3D). This analysis revealed that most cells in the lymph node have a fast-moving behavior compared with splenic lymphocytes. Cells in the white pulp are mostly crawling, while red pulp cells are predominantly sessile (Figure 3E). To further delineate lymphocyte behavior in the splenic white pulp specifically, we performed the same workflow for tracks from white pulp images only (Figures 1F–1H; Figure S2B; Video S4). This analysis revealed that the majority of cells in the white pulp exhibited a meandering behavior (Figures 3F–3H). Next, we co-transferred polyclonal GFP-expressing T cells and tdTomato-expressing B cells into the mice and imaged the spleens for longer time periods (Figure 3I). This revealed that T cells migrated in the white pulp with a higher average velocity than B cells (T cell mean: 6.81 μm/min; B cell mean: 4.87 μm/min). The cells maintained this motility for at least 100 min of spleen imaging (Figure 3J and Video S5). Behavioral analysis of T and B cells using HMM and clustering (Figures S2C and S2D) identified that both cells employ directed, meandering, and scanning cell behaviors, but a greater proportion of B cells underwent a scanning mode of motility (Figure 3K). Therefore, motile lymphocytes in the white pulp employed a spectrum of migration modes to navigate this complex microenvironment.

Video S4. Behavioral clusters after lymphocyte tracking, HMM state extraction, and Leiden clustering, related to Figure 3

Video S5. 3-Photon imaging of polyclonal GFP^+^ T cells and tdTomato^+^ B cells in the splenic white pulp, related to Figure 3

### Cell migration behavior in the white pulp is guided by the stromal architecture

We observed that SHG and THG imaging of the spleen enabled label-free visualization of the collagen structures and central arteries, respectively, in the white pulp as well as in the lymph node (Figure 4A). We next asked if the cellular behavioral modes observed in the white pulp were influenced by the networks of collagen fibers (and the stromal cells that produce and surround these fibers). Therefore, we segmented SHG as a branching network to extract directionality patterns and the previously analyzed cellular behavior in relation to these patterns (Figure 4B). The anisotropy of the SHG branching network was quantified and related to the cell behavioral clusters (Figure 4C). This uncovered that in T cell regions with high SHG anisotropy, a higher proportion of cells underwent a directed, or high speed, high straightness mode of migration (Figure 4D). Thus, the behavior of lymphocytes was influenced by the surrounding spleen structure, particularly where the collagen network was highly aligned. It may be important to consider this relationship in the spleen to deduce cellular behaviors and responses during disease.

**Figure 4:**
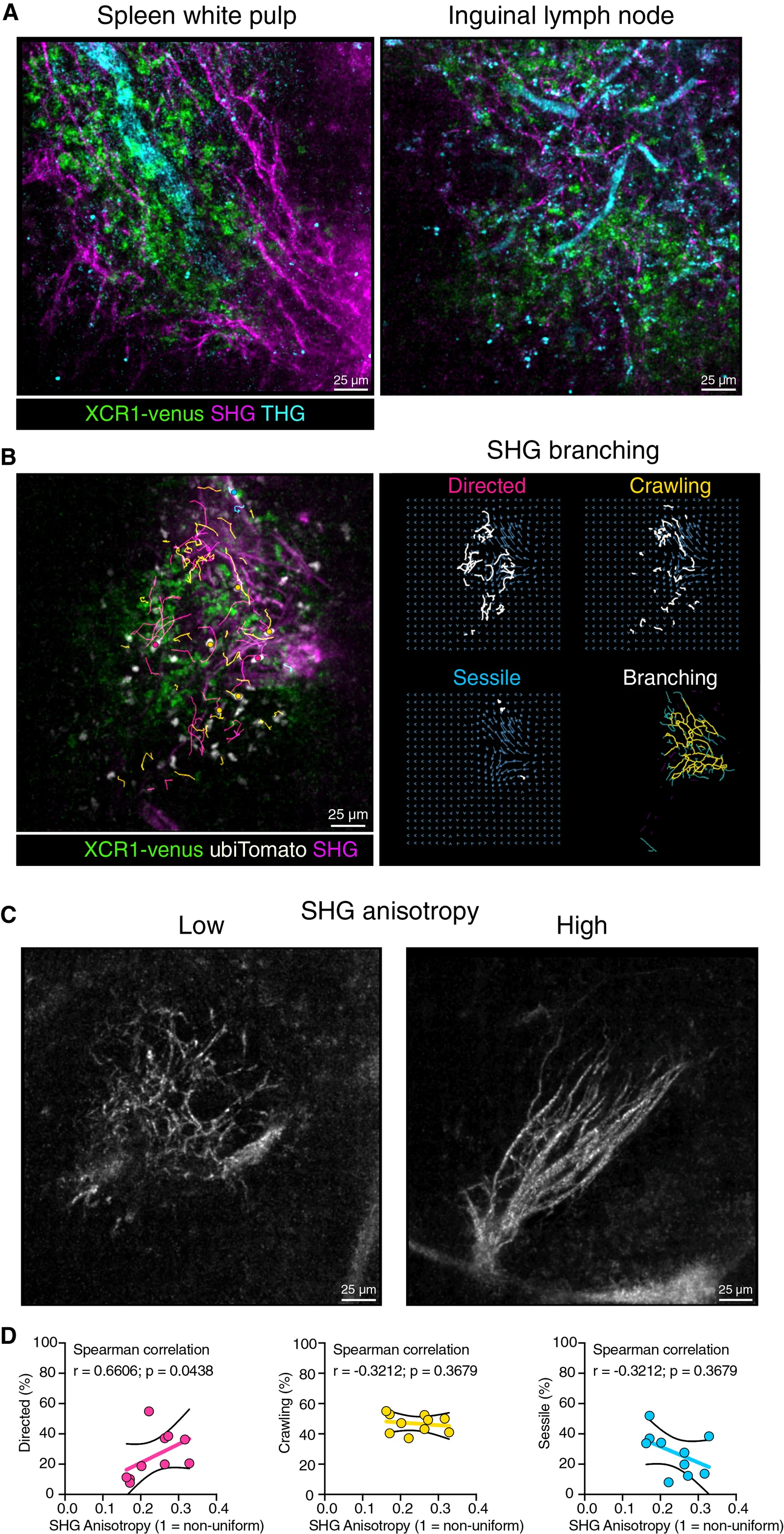
SHG and THG imaging reveals leukocyte migration behaviors in relation to the structural tissue components (A) Third harmonic generation (THG) signal from 3-photon microscopy. Examples depicting major blood vessels in the white pulp of the spleen (Video S6) and lymph node. (B) Branching networks of the second harmonic generation (SHG) signal depicted by directional flow and behavioral clusters (Figure 3F) overlayed. Color coding for SHG branching: junction-to-junction, yellow; tip-to-junction, green; tip-to-tip, purple. (C) Examples of white pulp regions with low- and high-SHG anisotropy. (D) SHG correlation with lymphocyte behavior. Quantified correlation between SHG anisotropy and lymphocyte behavior. *n* = 5 mice, 1–3 videos each, Spearman’s correlation.

## Discussion

We present a method to perform stable intravital imaging of the mouse spleen using 3-photon microscopy. We show that the construction of an imaging platform enables isolation of the spleen to stabilize the tissue and reduction motion from respiration. Our method is relatively simple, and the surgical intervention required to exteriorize the spleen is minor, which preserves spleen physiology for several hours of imaging.

We show that 3-photon imaging of the spleen prepared using this method enables reliable visualization of the white pulp. Although 2-photon imaging could be used with this method, 2-photon excitation provided limited access to the white pulp due to the depth of most white pulp regions in the murine spleen. 3-Photon excitation can be more sensitive to tissue heating and phototoxicity in moving samples because it requires higher excitation intensities, and motion can increase cumulative light exposure and energy deposition leading to enhanced photodamage. Our method enables isolation and stabilizing of the spleen to reduce movement during imaging and provides a protocol for imaging the splenic white pulp. Due to the repetition rate of the 3-photon laser, imaging is inherently slower than 2-photon excitation, enforcing limitations in imaging volume and the interval between images. For 3-photon imaging, we utilized a higher plane spacing and thinner imaging volume compared with 2-photon microscopy to achieve similar timelapse intervals. Reduced averaging was also used to speed up imaging, and we applied a denoising protocol to improve subsequent image quality for segmentation and cell tracking. Development and application of multi-plane imaging, such as reverberation multi-photon microscopy,^17^ could be used in the future to enable faster volumetric 3-photon imaging.

Our experiments using 3-photon imaging of lymphocytes in the spleen highlighted the disparity in motility between cells in the red pulp versus the white pulp. Cells in the red pulp were largely immotile, whereas cells migrating in splenic T cell zones moved rapidly and displayed an amoeboid cell morphology. This is consistent with a prior report^6^ and is likely due to the abundance of chemotactic signals in the white pulp, including the CCR7 ligand CCL21. Notably, when we compared T cell migration in splenic white pulp with that in the inguinal lymph node, we found that the speed of cell movement was slower in the spleen under the same imaging conditions. Because multiple soluble and receptor-mediated signals together contribute to the speed of cell migration in different tissue environments,^18^^,^^19^ this suggests that different signals or amounts of the same signals may influence cell motility in the spleen as compared with lymph nodes.

We performed behavioral analysis on 3-photon imaging data of the migrating lymphocytes using the Cecelia package.^16^ Previously published studies^20^^,^^21^ that aim to classify behavior using unsupervised clustering have mostly relied on summarized tracking statistics for movement and morphology. While this approach can identify gross behavioral differences, it does not consider the movement transitions within each track. With Cecelia, we utilized HMM states, which can recognize time-dependent morphology and movement sequences that are not captured by summarized tracking statistics. Using HMM states and their transitions within each track, we can more clearly identify behavioral differences.^16^

Our experiments revealed that most cells crawl or move in a rapid directional motion within the white pulp. Using SHG imaging, we revealed the collagen-rich stromal network in the T cell zone of the spleen. We found that lymphocytes use this branching network to guide their migration, consistent with explant imaging of cells in the spleen.^22^ Notably, cells undergoing directed motion were significantly influenced by the stromal microenvironment, whereas crawling cells, which may be responding to other signals, were not influenced by the anisotropy of the stromal architecture. We observed less SHG in lymph nodes by 3-photon microscopy, suggesting the possibility that the different structures of the collagen-rich stromal microenvironment in the splenic white pulp might influence the motility of lymphocytes. Further work is required to dissect cell migration in spleen, but the method described herein provides a firm basis for significant advances in this area.

### Limitations of the study

Our spleen mounting method for intravital imaging does not require a 3-photon microscope, but we find that reliable imaging of the splenic white pulp substantially benefits from the longer wavelengths used for 3-photon excitation. In addition, while 3-photon intravital microscopy can achieve more depth than 2-photon microscopy, there is a higher risk of tissue heating due to the high average power required for 3-photon excitation. Deviations in tissue position due to drift can result in increased laser power at the sample and lead to cells stopping or visible tissue deformation (Video S6), as previously described in lymph nodes.^12^ Our imaging platform isolates the spleen for more stable imaging, but movements in the tissue can still occur and must be accounted for when imaging. We have not tested this method during infections where there may be splenomegaly that may make the procedure more difficult due to increases in spleen size or vascularity.

Video S6. Examples of spleen movement during imaging that can lead to cells stopping or tissue phototoxicity due to phototoxicity, related to Figure 3

## Resource availability

### Lead contact

Requests for resources of this article and any additional information should be directed to the lead contact, Scott N. Mueller (smue@unimelb.edu.au).

### Materials availability

This study did not generate new unique reagents.

### Data and code availability

• All data reported in this paper will be shared by the lead contact upon request.
• This paper does not report original code.
• Cecelia can be accessed at https://github.com/schienstockd/cecelia
• Any additional information required to reanalyze the data reported in this paper is available from the lead contact upon request.

## Acknowledgments

This work was supported by a National Health and Medical Research Council Investigator Fellowship to S.N.M. (2017220) and the 10.13039/501100000923Australian Research Council (grant LE210100001). We thank the Biological Optical Microscopy Platform at the University of Melbourne for providing access to microscope facilities and the Biological Research Facility for management, breeding, and maintenance of mice. This research was supported by The University of Melbourne’s Research Computing Services and the Petascale Campus Initiative.

## Author contributions

D.S. and S.N.M. conceived the project; D.S. and S.D. performed experiments; D.S. performed data analysis, computational implementation, and software development; D.S. wrote the manuscript; D.S. and S.N.M. edited the manuscript; S.N.M. supervised the entire project.

## Declaration of interests

The authors declare no competing interests.

## STAR★Methods

### Key resources table

**Table:** 

REAGENT or RESOURCE	SOURCE	IDENTIFIER
**Chemicals, peptides, and recombinant proteins**		

Agarose	Bioline	Cat#: BIO-41025-BL
Molykote high-vacuum silicone grease	Dow Corning	Cat#: z273554-1ea
Isoflurane	Pharmachem	N/A
Tissue Adhesive Skin Glue	ZebraVet	Cat#: VEG3

**Experimental models: Organisms/strains**		

ubiTomato mice	Devi et al.^14^	N/A
UBC-GFP mice	Devi et al.^14^	RRID#: IMSR_JAX:004353
XCR1-Venus mice	Yamazaki et al.^23^	MGI:5544058

**Software and algorithms**		

Cecelia	Schienstock et al.^16^	https://github.com/schienstockd/cecelia
Cellpose	Stringer and Pachitariu^15^	https://github.com/mouseland/cellpose
btrack	Ulicna et al.^24^	https://github.com/quantumjot/btrack
skan	Nunez-Iglesias et al.^25^	https://github.com/jni/skan
ILEE	Li et al.^26^	https://github.com/phylars/ILEE_CSK
ThorImage	Thorlabs	Version 4.4
Graphpad Prism	Graphpad	Version 11

**Other**		

White Dwarf dual 3-photon laser	Class5	N/A
35 mm TC-treated Easy-Grip Style Cell Culture Dish	Falcon	Cat#: 353001
Advanced A-PAP Pen Mini	Daido Sangyo	Cat#: DAI-APAP-M
Change-A-Tip High Temperature Power Handle	Bovie Medical	Cat#: HIT1
Coverslips 24 × 24 mm	Knittel	Cat#: G410
DC Temperature Controller System	FHC	Cat#: 40-90-8D-01
Durapore Surgical Tape	3 M	Cat#: TRM00435
Gauze Swab 5 × 5 cm	Sentry Medical	Cat#: GS004
Gauze Swab 7.5 × 7.5 cm	ZebraVet	Cat#: PZ138-XXX
Heating Pad	FHC	Cat#: 40–90-2-02
Objective 25× water immersion	Olympus	Cat#: XLPN25XWMP2
Super Trimmer	Wahl	Cat#: 1592
Surgical Scalpel Blade	Swann-Morton	Cat#: 0210
Thorlabs Bergamo II multiphoton microscope	Thorlabs	N/A
Water for Irrigation	Baxter	Cat#: AHF7114

### Experimental model and study participant details

Animal experiments followed the NHMRC Code of Practice for the Care and Use of Animals for Scientific Purposes guidelines and were approved by the University of Melbourne Animal Ethics Committee (project 25292). XCR1-venus,^23^ ubiTomato^14^ and UBC-GFP (C57BL/6-Tg(UBC-GFP)30Scha/J) mice were bred and housed in specific pathogen-free conditions in the Biological Research Facility (BRF) at the Doherty Institute for Infection and Immunity, the University of Melbourne. Female mice, 6–10 weeks old, were used for all experiments. All animals were kept in HEPA filtered, individually ventilated cages, 19–22 C, 40–70% humidity, with environmental enrichment, 12 h light dark cycle and food and water *ad libitum*.

### Method details

#### Lymphocyte enrichment and adoptive transfer

T- and B-lymphocytes were negatively enriched from LN and spleens of UBC-GFP mice and ubiTomato mice, respectively. Recipient mice received an intravenous adoptive transfer of 6 × 10^6^ total cells, comprising two distinct populations combined at a 1:1 ratio, in preparation for intravital imaging.

#### Multiphoton microscope setup

We utilized a custom-made Thorlabs Bergamo II multiphoton microscope and ThorImage version 4.4 with super broadband optics for imaging from 680 to 1900nm and a Class 5 White Dwarf dual output 3-photon laser, with tuneable 1300nm and 1700nm laser lines. We utilised this setup to capture cell dynamics in the splenic white pulp and the inguinal lymph node. 2-photon microscopy commonly used for intravital imaging will only reveal the first 100–150 μm with exponential decrease in signal quality which will increase the user’s dependence on chance to find a very shallow white pulp. With 3-photon microscopy we found that there were frequently sufficient (more than 5) clear white pulp regions accessible to imaging. In addition, 3-photon excitation generated SHG is essential to navigate the splenic landscape. Regions high in collagen fibers (collagen nests) identify white pulp regions, whereas areas high in autofluorescence and no clear collagen nests are red pulp.

#### Preparation for mouse surgery

The custom-made imaging platform with adjustable stage was produced from stainless steel sheets but can be made from other suitable materials. The adjustable stage has two screws with a spring and a thin metal plate on top. A tissue culture dish with an approximately 9 mm hole cut out of the center was used to adhere and stabilise the spleen. We used a standard 35 mm round tissue culture dish and that was prepared with Durapore Medical tape (3 M) to avoid glueing the spleen directly onto the dish. Before beginning the surgery, melt 2% agarose in PBS with a microwave and keep at 35°C–40°C until needed. Before closing the portal, transfer the agarose jar onto ice to cool down until warm to touch and then apply to the spleen. Otherwise, the heated agarose might cause adverse effects due to the increased temperature. Ensure that the heat pad is warmed up to ∼37°C before starting the surgery.

#### Spleen surgery

During the surgery, it is advisable not touch the spleen and to keep the tissue hydrated with PBS. Handling of the spleen can lead to bleeding and will either lead to reduced imaging quality or damage to the tissue and stopping of the cells to image due to blood loss. Scissors should be sufficiently sharp to enable accurate surgery. We provided a video for the whole procedure to follow the individual steps (Video S1).

Induce anesthesia with an approved method. We induced anesthesia with 2% isoflurane and placed the mouse on a heat pad with nose cone to maintain anesthesia (Figure 1Di). Shave the left flank with an electric razor (Figure 1Dii) and wash with ethanol to capture removed hair. Using forceps, stretch skin on the left flank above the approximate region of the spleen and cut along the direction of the spleen, caudal-to rostral, with surgical scissors (Figure 1Diii). Cut through the peritoneum and muscle layer over spleen and use a cauteriser to seal the cut site. Place PBS-soaked gauze over the cut area with an opening over the incision site (Figure 1Div).

Transfer the mouse to the metal platform and place this back on heat pad. Lightly tighten the screws to position the stage and mouse (Figure 1Dv). Push the stage down just enough to cause the spleen to be slightly externalised and use forceps to gently expose the spleen (Figure 1Dvi). The stage does not need to be very tight, just enough for the spleen to be exposed. If the stage is too tight, it will restrict breathing of the mouse due to diaphragm constriction. This is not good for the mouse and not ideal for imaging as it causes increased movement due to breathing.

Adhere the tissue culture dish to the stage with four spots of vacuum grease (Figure 1Dvi, pink dots). Carefully guide the spleen through the portal with forceps or a PBS-moistened cotton tip using the pancreas (Figure 1Dvii). Avoid directly touching the spleen as this can cause tissue damage and bleeding. For increased stability, the tip of the spleen can be adhered to the tape on the dish with tissue adhesive on the underside of the tissue. Only use sufficiently enough glue for the spleen to stay in place. At this stage it is important to not place the spleen on an angle on the pancreas or underlying tissue. This will significantly reduce the tissue area to be imaged. The spleen should be flat, either directly on the pancreas or next to it but not half on and half off. The coverslip should create an evenly spread contact area. An uneven imaging area will result in uneven plane illumination potentially causing tissue heating and phototoxicity.

Create a grease border around the spleen on culture plate, taking care not to get any grease onto the spleen (Figure 1Dviii). Place agarose on ice to cool down but remain fluid (Figure 1Dix). Cut off the tip of a pipette to make agarose pipetting easier (Figure 1Dx) and fill the portal of the tissue culture dish with agarose to close it off. Use minimal agarose, just enough to close the portal. Otherwise, it can lead to an angled coverslip as agarose, once hardened, cannot be compressed. This might also obstruct a flush interface between the spleen and the coverslip. Then submerge in warmed PBS (Figure 1Dxi). Place a coverslip on top of grease border and carefully push down with forceps to slightly flatten the spleen against the coverslip. Do not compress the spleen too much as this could affect cell motility. It is ok if the grease barrier is a bit leaky, as long as it sealed once the pressure of the coverslip is applied. Draw a hydrophobic border onto the coverslip around the spleen. Do not draw over the imaging field.

Move the mouse on the imaging platform to the microscope and ensure anesthesia is maintained (Figure 1E). Add distilled water onto the coverslip and immerse the objective for imaging. Wait 1 h before starting imaging for the tissue to settle down. Slight drifting in Z-direction can cause tissue heating which in turn can exacerbate z drift. Tissue drift and damage can be detected by visible holes in the signal.^13^ A more subtle effect of tissue heating is that cells may stop, or the tissue distorts from heating (Video S5). Depending on the microscope, the detectors can also be triggered due to saturated signal intensity.

#### Inguinal lymph node prep

Inguinal lymph nodes were prepared as described elsewhere.^4^ In brief, mice were shaved on their belly and left hind flank, and remaining hair was removed using Veet. The inguinal lymph node was exposed by creating a ‘tissue flap’ which was adhered to a custom-made block. Fat surrounding the lymph node was carefully removed with fine scissors and forceps. The flap was surrounded with grease to create a PBS-pool which was closed off with a coverslip.

#### Image acquisition

We utilised 2 MHz 3-photon excitation for imaging at 1300 nm as this provided us with the ability to capture cell dynamics in the white pulp which requires an imaging depth of around 100–300 μm (Figure 2A, right panel). 2-photon excitation could not be used reliably beyond 100 μm (Figure 2A, left panel). Images were acquired with 2× frame averages, a 30 s interval as a stack with 13 z planes and 3 μm intervals between planes. The imaging speed in 3-photon is significantly slower than in 2-photon and depends on the repetition rate of the laser beam. 2-photon excitation at 80 MHz was performed with 4× frame averaging, 20 s interval for stacks of up to 50 z planes and 2 μm intervals between planes.

#### Image analysis

The resulting image were processed using Cecelia.^16^ Images were imported as OME-Zarr and denoised using Cellpose^15^ within Cecelia to reduce noise and inhomogeneity within frames. Denoised images were corrected for autofluorescence, based on channel division, and drift with skimage’s phase cross correlation (https://scikit-image.org/docs/stable/api/skimage.registration.html). Cells were segmented using Cellpose,^27^ with a diameter of 10 μm, and tracked using btrack,^24^ with a maximum search radius of twice the cell diameter (20 μm).

Cell behavior was extracted using Hidden Markov Models (HMM) and unsupervised Leiden clustering. HMM states were extracted based on speed and angle. For combined analysis of lymph node and spleen, 5 HMM states were utilised and 4 HMM states for the white pulp only analysis. HMM states, their transitions and whole track measurements were fed into Leiden clustering with a resolution of 0.4 for combined tissue analysis and 0.3 for white pulp only analysis. The resulting clusters were further grouped into behavior types based on their track properties.

To analyze cell movement in relation to tissue structures, SHG was segmented using Cellpose with a small diameter. This segmentation was binarized within Cecelia’s branching module to create a network representation of these collagen structures based on the python library skan.^25^ The orientation/anisotropy of this branching network was further analyzed using ILEE.^26^

### Quantification and statistical analysis

Graphs and statistics were generated using Prism 11 (GraphPad). Samples were tested for normality. Multiple groups were analyzed with one-way ANOVA and Tukey’s multiple comparison test. All graphs depict means ± SEM. Details of statistical analysis are indicated in the figure legends and include the statistical test used with respective *p*-values in the figures. Interaction between behavior and SHG anisotropy was tested for significance with Spearman correlation.

## Supplemental information

Document S1. Figures S1 and S2

Document S2. Article plus supplemental information
