# Supplementary material for: Intravital 3-photon microscopy of leukocyte dynamics in the splenic white pulp
Source: Cell Rep Methods. 2026 Sep 25;6(10):101609. doi: 10.1016/j.crmeth.2026.101609 (PMC13721799; doi:10.1016/j.crmeth.2026.101609)
Supplement: Supplementary file 1 [file mmc1.pdf]

Cell Reports Methods, Volume *6*

Supplemental information

Intravital 3-photon microscopy of leukocyte dynamics in the splenic white pulp

Dominik Schienstock, Sapna Devi, and Scott N. Mueller

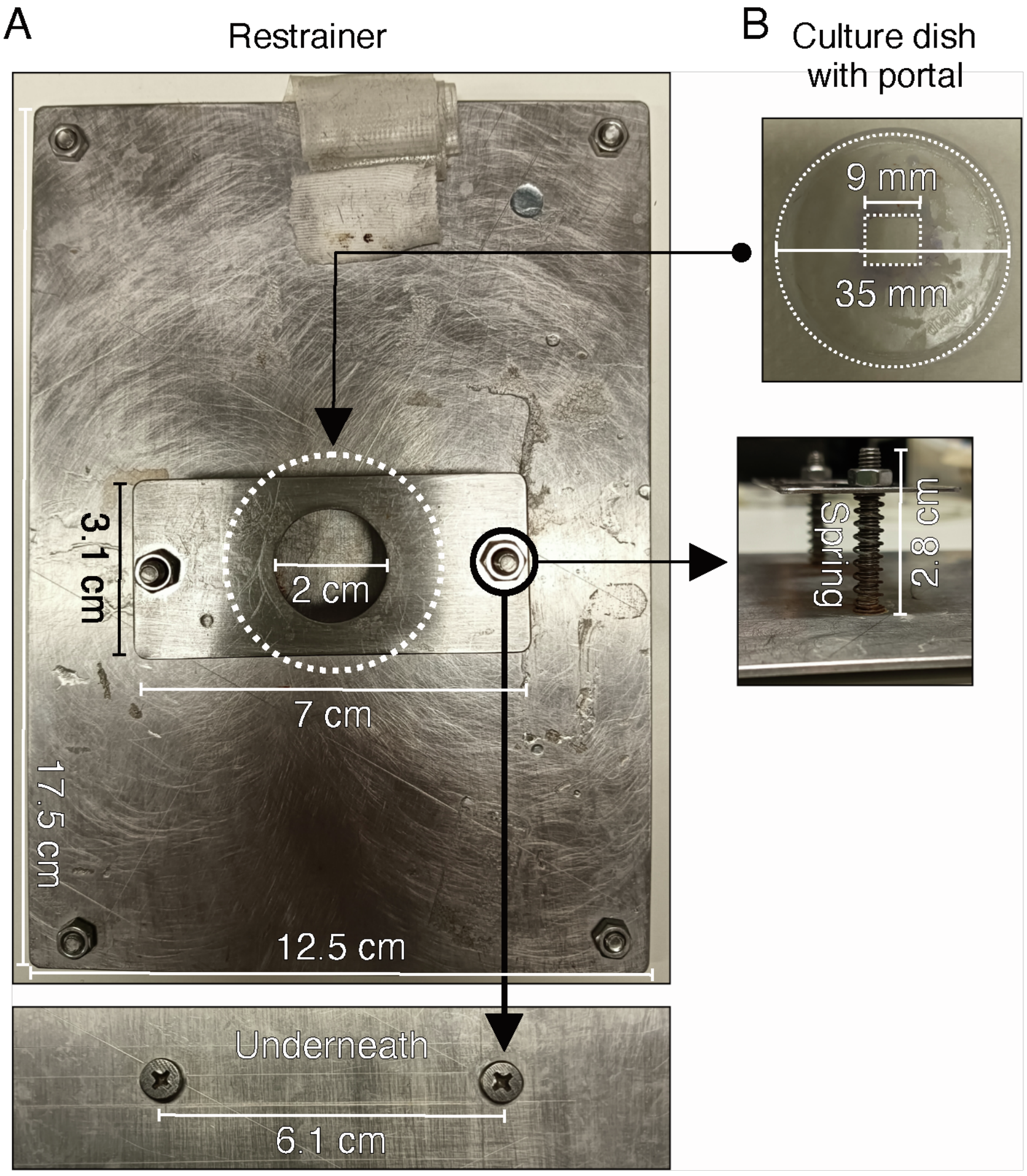

Figure S1.

Design and dimensions for the spleen imaging platform. Related to Figure 1. **(A)** and prepared tissue culture dish **(B)**. The thickness of the base plate is 1 mm and of the adjustable stage 0.5mm.

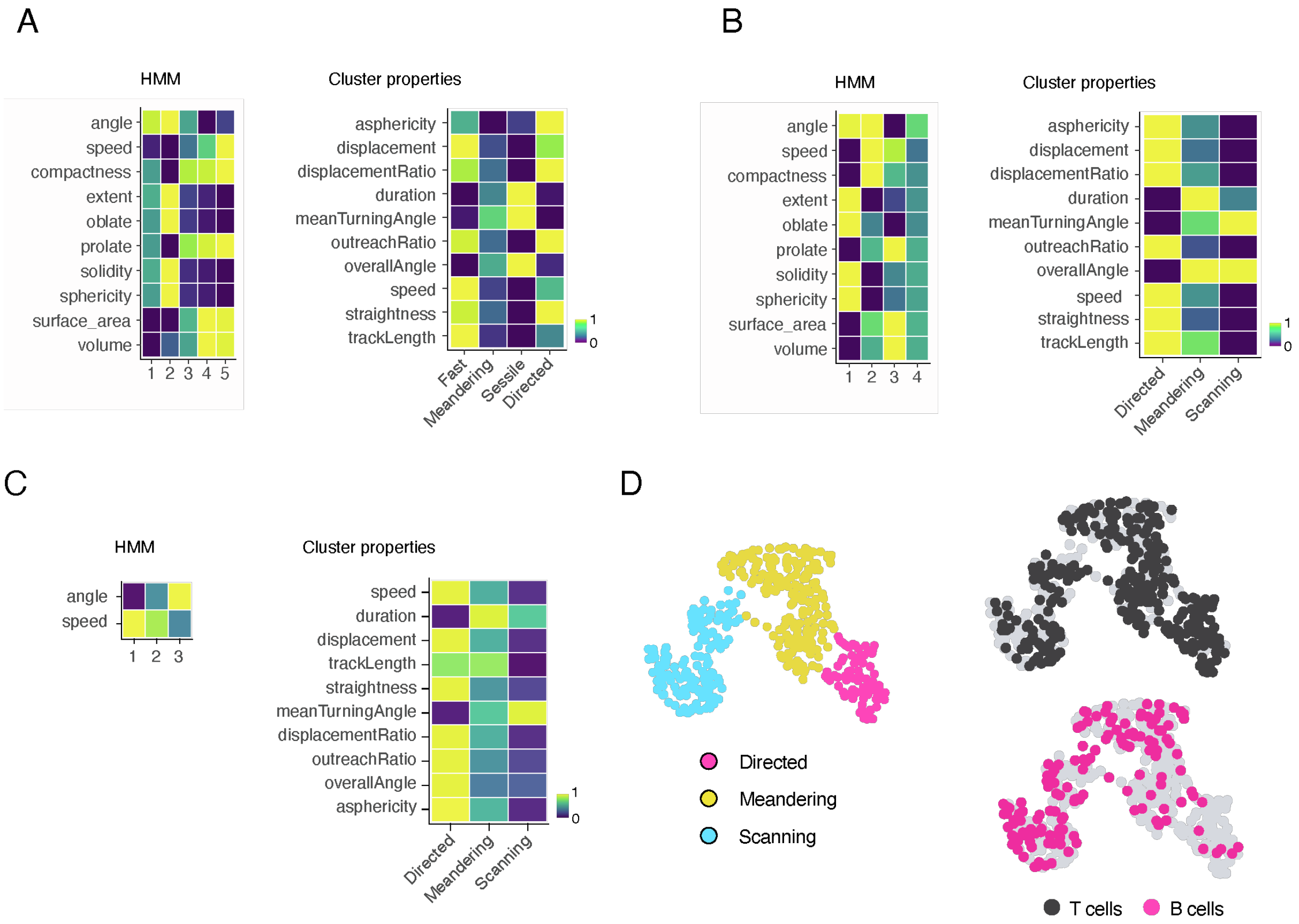

Figure S2.

HMM analysis of lymphocytes imaged in the spleen and lymph nodes by 3-photon microscopy. Related to Figure 3.

(A) HMM and cluster properties for combined analysis of lymph node and spleen locations (related to Fig. 3C-E).

(B) HMM and cluster properties for white pulp only analysis (related to Fig. 3F-H).

(C) HMM and cluster properties for white pulp only analysis (related to Fig. 3I-K).

(D) Resulting UMAP based on Leiden clustering using HMM, HMM transitions and whole track measurements. UMAP projections demonstrate behavioural clusters (left) and cell type distribution (right).
